# Differential activation of CD57-defined natural killer cell subsets during recall responses to vaccine antigens

**DOI:** 10.1111/imm.12239

**Published:** 2014-04-10

**Authors:** Matthew J White, Carolyn M Nielsen, Reuben H C McGregor, Eleanor M Riley, Martin R Goodier

**Affiliations:** Department of Immunology and Infection, Faculty of Infectious and Tropical Diseases, London School of Hygiene and Tropical MedicineLondon, UK

**Keywords:** CD57, diphtheria–tetanus–pertussis vaccine, natural killer cells

## Abstract

Natural killer (NK) cells contribute to the effector phase of vaccine-induced adaptive immune responses, secreting cytokines and releasing cytotoxic granules. The proportion of responding NK cells varies between individuals and by vaccine, suggesting that functionally discrete subsets of NK cells with different activation requirements may be involved. Here, we have used responses to individual components of the DTP vaccine [tetanus toxoid (TT), diphtheria toxoid (DT), whole cell inactivated pertussis] to characterize the NK cell subsets involved in interleukin-2-dependent recall responses. Culture with TT, DT or pertussis induced NK cell CD25 expression and interferon-*γ* production in previously vaccinated individuals. Responses were the most robust against whole cell pertussis, with responses to TT being particularly low. Functional analysis of discrete NK cell subsets revealed that transition from CD56^bright^ to CD56^dim^ correlated with increased responsiveness to CD16 cross-linking, whereas increasing CD57 expression correlated with a loss of responsiveness to cytokines. A higher frequency of CD56^dim^ CD57^−^ NK cells expressed CD25 and interferon-*γ* following stimulation with vaccine antigen compared with CD56^dim^ CD57^+^ NK cells and made the largest overall contribution to this response. CD56^dim^ CD57^int^ NK cells represent an intermediate functional phenotype in response to vaccine-induced and receptor-mediated stimuli. These findings have implications for the ability of NK cells to contribute to the effector response after vaccination and for vaccine-induced immunity in older individuals.

## Introduction

Natural killer (NK) cells are classically regarded as a stable population of innate immune effectors that, by cytokine production or cytotoxicity, help to contain an infection or limit tumour growth until an effective adaptive response is mounted. However, numerous lines of evidence now suggest that NK cells adapt functionally after stimulation by viruses, cytokines and hapten antigens; this phenomenon has been termed ‘NK memory’ but may also reflect functional NK cell maturation.^1^ There are several routes by which NK cell function may be enhanced during re-exposure to a pathogen. Antigen-specific memory T cells secreting interleukin-2 (IL-2) promote NK cell function and proliferation, while pathogen-specific antibodies initiate antibody-dependent cellular cytotoxicity by cross-linking CD16 or other Fc receptors for immunoglobulins.^2^–^6^ Alternatively, cytokines released during primary infection may induce NK cells to proliferate and/or differentiate to a more highly responsive state; subsets of NK cells expressing activating receptors able to bind specific pathogen ligands may be particularly responsive (as described for the Ly49H^+^ subset of mouse NK cells which bind the murine cytomegalovirus m157 protein^7^).

A number of NK cell subsets with different functional potential have now been described in humans. The least mature circulating NK cells are CD56^bright^ CD57^−^ and are assumed to give rise to CD56^dim^ CD57^−^ cells which, in turn, mature into CD56^dim^ CD57^+^ cells, the latter subset increasing in frequency with increasing age.^8^,^9^ This three-step maturation is associated with acquisition of CD16, CX3CR1, granzyme and KIR, gradual loss of proliferative capacity, reduced responsiveness to cytokines such as IL-12 and IL-18, and increasing cytotoxic function.^10^,^11^ CD56^dim^ CD57^+^ NK cells express lower levels of IL-18R*α*^10^ as well as lower levels of mRNA for the inducible chain of the IL-12R (IL-12R*β*2)^12^ suggesting that these NK cells may respond less well than other subsets to IL-12 and IL-18. Conversely, CD56^dim^ CD57^+^ cells express higher levels of CD16, suggesting that they may be particularly good mediators of antibody-dependent cellular cytotoxicity.^12^

The potential for NK cells to respond to exogenous cytokines is central to their ability to control infections,^4^,^13^,^14^ particularly where ligands for other NK-activating receptors are lacking. Moreover, NK cells responding to CD4^+^ T-cell-derived IL-2 have the potential to contribute to secondary immune responses, including those induced by vaccination.^3^,^4^ We wondered, therefore, whether NK cell subsets would differ in their ability to mount ‘recall’ responses to vaccine antigens. To test this hypothesis, we have assessed the capacity of various NK cell subsets, defined principally by their expression of CD56 and CD57, to contribute to a recall response to the components of diphtheria–tetanus–pertussis (DTP) vaccine. We find that vaccine-induced NK cell interferon-*γ* (IFN-*γ*) and degranulation (CD107a) responses differ between NK cell subsets. Importantly, our studies reveal that CD57 expression is gained in a gradual stepwise fashion and that changes in NK cell function mirror this progressive maturation.

## Materials and methods

### Donors and peripheral blood mononuclear cell preparation

Volunteers were recruited from among staff and students at the London School of Hygiene and Tropical Medicine. All subjects gave fully informed, written consent and the study was approved by the London School of Hygiene and Tropical Medicine Ethics Committee. Subjects ranged in age from 21 to 73 years and all donors confirmed that they had been vaccinated against diphtheria, tetanus and pertussis in childhood. Peripheral blood mononuclear cells (PBMC) were separated by fractionation on a Ficoll–Hypaque gradient and cryopreserved in liquid nitrogen. Frozen PBMC were thawed with pre-warmed complete medium [RPMI-1640 supplemented with 100 U/ml penicillin/streptomycin and 20 mm l-glutamine (Gibco, Lifesciences, Paisley, UK) and 10% pooled human AB serum (Sigma, Poole, UK)] (at 37°), washed several times and rested for 30 min before use.

### NK cell assay culture

Peripheral blood mononuclear cells (2 × 10^5^ cells in 200 μl) were cultured in 96-well U-bottom plates in complete medium with or without low concentration of cytokines [LCC; 12·5 pg/ml recombinant human (rh) IL-12 (PeproTech, Rocky Hill, NJ) plus 10 ng/ml rhIL-18 (MBL, Woburn, MA)]; high concentration of cytokines (HCC; 5 ng/ml rhIL-12 plus 50 ng/ml rhIL-18); or 7·5 μg/ml tetanus toxoid (TT), 1 μg/ml diphtheria toxoid (DT) or 1 IU/ml whole cell pertussis (all from the National Institute for Biological Standards and Control, London, UK) for 18 hr at 37°. GolgiPlug (containing Brefeldin A, 1/1000 final concentration; BD Biosciences, Oxford, UK) and GolgiStop (containing Monensin, 1/1500 concentration; BD Biosciences) were added after 15 hr.

### Receptor cross-linking

Flat-bottomed 96-well plates were coated (overnight at 4°) with 50 μl of mouse monoclonal antibody to human CD16 (final concentration of 20 μg/ml; BD Biosciences) or a cocktail of monoclonal antibodies to human NK receptors [NKG2D, NKp30, NKp46, 2B4 (all from R&D Systems, Abingdon, UK)] and CD2 (BD Biosciences) at an overall combined concentration of 20 μg/ml, i.e. 4 μg/ml each. An equivalent concentration of mouse IgG1 *κ* isotype control antibody (BD Biosciences) was used as a negative control. After washing (three times in sterile PBS), 2 × 10^5^ PBMC were added to each well and incubated for 18 hr. GolgiPlug and GolgiStop were added after 15 hr. Cells were then transferred to 96-well U-bottomed plates for washing and staining.

### Flow cytometry

Responses of NK cells and T cells were assessed as described previously.^15^ Briefly, cells were stained with fluorophore-labelled monoclonal antibodies to cell surface molecules, fixed, permeabilized and stained for intracellular molecules using a Cytofix/Cytoperm kit (BD Biosciences). Cells were analysed by flow cytometry on an LSR II (BD Biosciences). Samples with fewer than 100 NK cells in each subset were excluded. The following reagents were used: anti-CD56-phycoerythrin (PE) -Cy7, anti-CD16-allophycocyanin (APC) -H7, anti-CD4-Pacific Blue, anti-IFN-*γ*-e780, anti-IFN-*γ*-APC, anti-CD3-V500 and anti-CD69-phycoerythrin-cyanine5 (PE-Cy5) (all BD); anti-CD8-PE-Cy5, anti-CD25-PE, anti-IL-18R*α*-PE, anti-CD62L-PE-Cy5, anti-CD57-e450 and anti-IL-2-APC (all e-Biosciences/Affimetrix, Hatfield, UK). Anti-IL-12R*β*2 monoclonal antibody was obtained from R&D Systems (Oxford, UK) and conjugated to PE-Cy5 using an Easylink PE/Cy5® Conjugation Kit (Abcam, Cambridge, UK).

### Data and statistics

Unless stated to the contrary, all figures show data from at least three replicate experiments. Flow cytometry data were analysed using Flow Jo (Tree Star, Ashland, OR) and data were analysed using Prism6 (GraphPad, San Diego, CA) software. Statistical comparisons were performed by paired analysis of variance or *t*-tests. Correlation between parameters was by bivariate regression analysis. ****P ≤ 0·0001, ***P < 0·001, **P < 0·01, *P < 0·05.

## Results

### DTP vaccination induces durable vaccine antigen-driven NK cell responses

**Figure 1:**
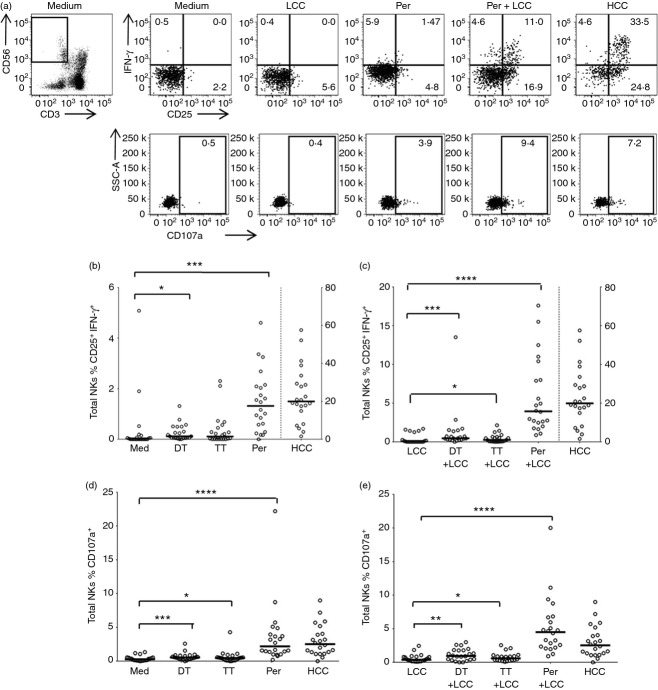
Natural killer (NK) cell responses to diphtheria toxoid (DT), tetanus toxoid (TT) and whole cell pertussis. Peripheral blood mononuclear cells (PBMC) from previously vaccinated donors were cultured *in vitro* for 18 hr with medium alone, low concentration of cytokines (LCC), DT, TT, pertussis (Per), DT + LCC, TT + LCC, Per + LCC, or high concentration of cytokines (HCC). (a) Representative flow cytometry plots showing gating of CD56^+^ CD3^−^ NK cells and expression of CD25, CD107a and interferon-*γ* (IFN-*γ*). (b, c) Percentage of NK cells co-expressing CD25^+^ and IFN-γ^+^ after stimulation in the absence (b) or presence (c) of LCC. (d, e) Percentage of NK cells expressing CD107a after stimulation in the absence (d) or presence (e) of LCC. Note: in (b) and (c) HCC data are shown on a different axis (see right hand side of plot). Each data point represents one donor, *n *= 22. Lines represent median values. Data were analysed with paired, non-parametric *t*-tests. **** *P *≤ 0·0001, *** *P *< 0·001, ** *P *< 0·01, * *P *< 0·05.

To validate DTP vaccination as a suitable model for evaluating NK cell recall responses, PBMC were incubated overnight with TT, DT or inactivated whole cell pertussis with or without low concentrations of the cytokines IL-12 and IL-18 (LCC) or, as a positive control, with a high concentration of cytokines IL-12 and IL-18 (HCC), stained for NK cell phenotypic and functional markers, and examined by flow cytometry (Fig. 1). HCC induces over 50% of CD3^−^ CD56^+^ NK cells to express cell surface CD25 and intracellular IFN-*γ* (median 19·9%, range 1·6–57·5, Fig. 1a–c) and has a significant, but much less marked, effect on CD107a expression (median 2·5%, range 0·001–9·0, Fig. 1a,d,e). By contrast, LCC alone induces a small, but significant, proportion of NK cells to express CD25 (median 6·4%, range 0·6–25·4), but few, if any, of these cells also produce IFN-*γ* (median 0·0%, range 0·0–1·68) or express CD107a (median 0·4%, range 0·1–2·4) on their surface (Fig. 1a).

Among PBMC stimulated with vaccine antigen alone (i.e. without LCC) there is highly significant up-regulation of both CD25 and IFN-*γ* by NK cells in response to pertussis (median 1·3%, range 0·0–4·6), a lesser (but still significant) response to DT (median 0·1%, range 0·0–1·3) and no significant response to TT (median 0·1%, range 0·0–1·3) (Fig. 1b). However, responses to all three antigens were significantly enhanced in the presence of LCC (pertussis: median 3·9%, range 0·9–17·6; DT: median 0·5%, range 0·0–13·5; TT: median 0·3%, range 0·0–2·13) (Fig. 1c) and were ablated in the presence of neutralizing antibody to IL-2 (data not shown). These data are fully consistent with a scenario in which a whole cell antigen such as pertussis contains ligands for Toll-like receptors^16^ and so induces accessory cells to secrete cytokines such as IL-12 and IL-18, whereas purified proteins such as TT and DT do not; exogenous LCC induces expression of CD25 (and so the high-affinity IL-2R) on NK cells allowing them to respond to IL-2 from vaccine-specific CD4^+^ T cells. By contrast, a statistically significant increase in CD107a expression on NK cells was seen in response to all three vaccine components (pertussis: median 2·2%, range 0·2–22·2; DT: median 0·5%, range 0·0–2·6; TT: median 0·5%, range 0·0–4·3) (Fig. 1d) and this was not significantly enhanced by LCC (pertussis: median 4·5%, range 0·9–20·0; DT: median 0·9%, range 0·0–3·0; TT: median 0·6%, range 0·1–2·5) (Fig. 1e).

### CD57 is a stable marker of human NK cell subsets

Despite very robust NK cell responses to some of the vaccine antigens, not all NK cells responded and there is considerable heterogeneity in the magnitude of the NK cell response between donors (Fig. 1b–e). Although heterogeneity between individuals might be explained by variation in the strength of the T-cell IL-2 response that drives the NK responses,^3^,^17^,^18^ this is unlikely to explain heterogeneity of responses within the NK cell population of an individual donor. We therefore considered whether within-donor variation might be the result of differences between subsets of NK cells in their intrinsic sensitivity to activation by monokines and T-cell-derived IL-2.

**Figure 2:**
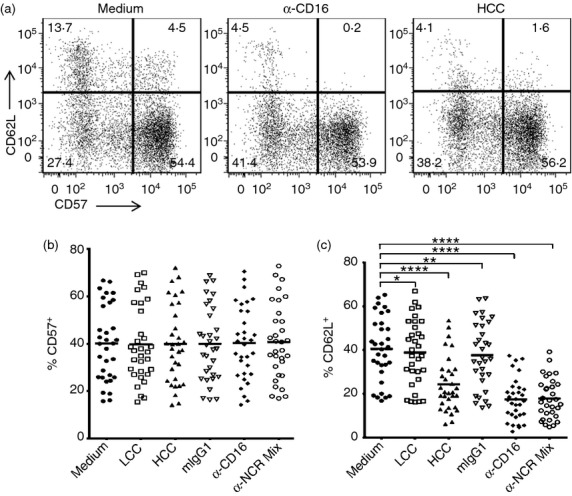
CD57 is a stable marker of human natural killer (NK) cell subsets. Peripheral blood mononuclear cells (PBMC) were cultured *in vitro* for 18 hr with plate-bound mouse IgG1 isotype control (mIgG1), anti-human CD16, anti-human NK cell receptor (NKR) cocktail (*α–*CD2, *α–*NKG2D, *α–*NKp30, *α*–NKp46) (all to a final concentration of 20 μg/ml), low concentration of cytokines (LCC) or high concentration of cytokines (HCC). (a) Representative flow cytometry plots showing expression of CD62L and CD57 on gated (CD56^+^ CD3^−^) NK cells. (b) Percentage of NK cells that were CD57^+^ after PBMC culture under different conditions. (c) Percentage of NK cells that were CD62L^+^ after PBMC culture under different conditions. *P*-values are derived from repeated measures analysis of variance (c). Each data point represents one donor, *n *= 31. Lines represent mean values. ****P ≤ 0·0001, **P < 0·01, *P < 0·05.

CD57 is a marker of highly differentiated, highly cytotoxic NK cells^12^,^19^,^20^ and CD62L (l-selectin) is a marker of cells able to proliferate and secrete IFN-*γ* after high-dose cytokine stimulation.^21^ However, to use these as markers of NK cell subsets in mixed PBMC assays, it was important to know whether they were stable phenotypic markers or whether their expression was altered after activation. To this end, expression of CD62L and CD57 were examined on PBMC after overnight stimulation with LCC or HCC, or with cross-linking antibody to the NK cell activating receptor CD16, or a cocktail of antibodies to NK cell activating receptors (NKp30, NKp46, NKG2D and CD2) (Fig. 2). Consistent with previous reports,^12^ CD62L and CD57 tended to define mutually exclusive subsets of NK cells (Fig. 2a). However, although CD57 expression appeared very stable after overnight activation by cytokines or receptor cross-linking (Fig. 2b), CD62L expression was markedly reduced after activation (Fig. 2c). Given the significant activation-induced down-regulation of CD62L, subsequent functional analysis of NK subsets was based on CD57 expression but not CD62L.

### CD56 and CD57 define multiple distinct NK cell subsets

**Figure 3:**
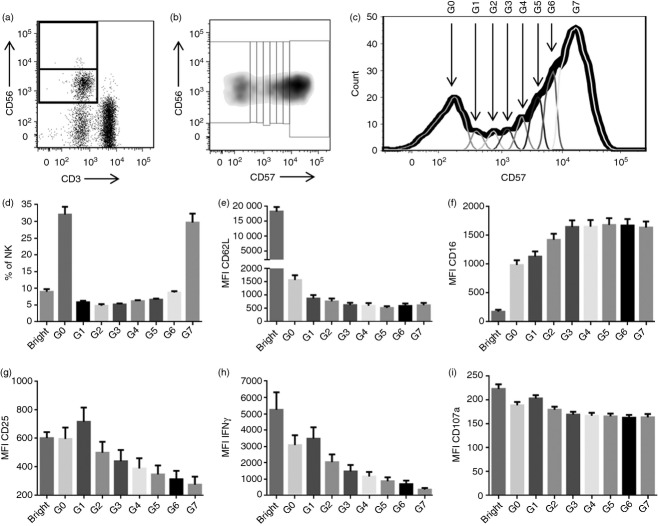
CD56 and CD57 define multiple distinct natural killer (NK) cell subsets. Representative flow cytometry plots showing gating of CD56^bright^ and CD56^dim^ NK cells (a) and corresponding dot plot (b) and histogram (c) showing gating of the CD56^dim^ subset into seven subpopulations based on CD57 expression. The G0 population represents CD56^dim^ CD57^−^ cells; G1–6 are CD56^dim^ CD57^int^ cells; G7 are CD56^dim^ CD57^+^ cells). (d–f) *Ex vivo* analysis of each subpopulation of NK cells (as defined in c) among NK cells from 32 donors: (d) mean (SEM) percentage of all NK cells which fall into each subpopulation; (e) mean (SEM) mean fluorescence intensity (MFI) of CD62L expression and (f) CD16 expression on each subpopulation. (g–i) Peripheral blood mononuclear cells from 32 donors were stimulated for 18 hr with high concentration of cytokines: mean (SEM) MFI of CD25 expression (g), interferon-*γ* (IFN-*γ*) expression (h), and CD107a expression (i) on each subpopulation. Bar charts represent means ± SEM, *n *= 32.

Expression of CD56 and CD57 has been used to identify three subsets of NK cells. Functional analysis of these subsets suggests that NK cells differentiate from relatively immature CD56^bright^ CD57^−^ cells, which respond to cytokine stimulation by producing IFN-*γ* but have limited cytotoxic potential, to CD56^dim^ CD57^−^ cells which are also poorly cytotoxic but retain IL-12R expression and so the ability the secrete IFN-*γ* in response to cytokine stimulation and, eventually, to CD56^dim^ CD57^+^ cells, which no longer respond to exogenous cytokines but are skewed towards a cytotoxic phenotype following cross-linking of CD16 or NK receptors or exposure to target cells.^10^,^12^,^20^ However, CD57 expression is not simply ‘off’ or ‘on’ but is gradually up-regulated in a stepwise fashion (Fig. 3). It was possible to identify seven distinct peaks of CD57 expression (Fig. 3b,c) with each peak accounting for ˜5% to ˜35% of all CD56^dim^ NK cells (Fig. 3d). CD62L expression is lost as soon as cells begin to express CD57 (Fig. 3e) but CD16 expression is gradually up-regulated, with maximal CD16 expression not being reached until the third peak of CD57 expression (Fig. 3f). Most importantly, the functional remodelling of NK cells, in terms of loss of cytokine-induced up-regulation of CD25 and IFN-*γ* expression, is extremely gradual with complete unresponsiveness to HCC not being seen until CD57 expression reaches its maximal level (Fig. 3g,h). By contrast, little or no difference was observed in the ability of NK cells with different levels of CD57 expression to degranulate in the presence of cytokines (Fig. 3i).

**Figure 4:**
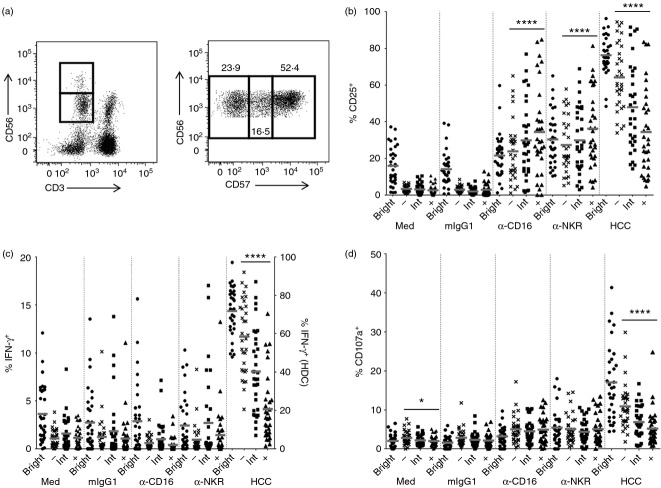
CD57 defines a continuum of functionally distinct natural killer (NK) cells. (a) Representative flow cytometry plots showing gating of CD56^bright^ and CD56^dim^ NK cells (left), and subsequent gating of the CD56^dim^ subset into CD56^dim^ CD57^−^, CD56^dim^ CD57 intermediate (CD57^int^) and CD56^dim^ CD57^+^ populations (right). (b–d) Peripheral blood mononuclear cells from 32 donors were cultured with cross-linking antibodies to CD16 or NK cell receptors, or with an isotype control mIgG1 or high concentration of cytokines (HCC), for 18 hr. Percentage of cells in each NK subset expressing CD25 (b), interferon-*γ* (IFN-*γ*) (c) and CD107a (d). Note: in (c), HCC data are shown on a different axis (see right hand side of plot). Each data point represents one donor, *n *= 33. Lines represent mean values. CD56^dim^ subsets were analysed for linear trend with a repeated measures analysis of variance. ****P ≤ 0·0001, *P < 0·05.

These data suggest that NK cells with intermediate levels of CD57 expression (CD57^int^), which represent a significant fraction (˜30%) of circulating NK cells, are also intermediate in terms of their functional maturation. To formally test this hypothesis, we analysed responses of the four NK cell subsets (CD56^bright^; CD56^dim^ CD57^−^; CD56^dim^ CD57^int^ and CD56^dim^ CD57^+^, Fig. 4a) to HCC, cross-linking of CD16 and cross-linking of NK receptors, by expression of CD25, IFN-*γ* or CD107a (Fig. 4b–d). As expected, high proportions of CD56^bright^ cells expressed CD25, IFN-*γ* or CD107a in response to HCC; cross-linking of CD16 or NK cell receptors up-regulated CD25 and CD107a but not IFN-*γ* in this subset (Fig. 4b–d). Among CD56^dim^ NK cells, CD25, CD107a and IFN-*γ* responses to HCC declined with increasing levels of CD57 expression with a statistically significant negative trend from CD56^dim^ CD57^−^ cells, through CD56^dim^ CD57^int^ cells to CD56^dim^ CD57^+^ cells (analysis of variance for all linear trends, *P *≤ 0·0001) (Fig. 4b–d). Interestingly, although no significant differences were observed between the three CD56^dim^ populations in their ability to degranulate or produce IFN-*γ* in response to CD16 or NK cell receptor cross-linking, the cross-linking of CD16 or NK cell receptors led to increasing levels of CD25 expression with increasing expression of CD57 (linear trend; *P *≤ 0·0001 in both cases), suggesting that responsiveness to T-cell IL-2 may be retained in CD57^+^ NK cells in the presence of antibodies able to induce antibody-dependent cellular cytotoxicity. In summary therefore, the transition from CD56^bright^ to CD56^dim^ (irrespective of CD57 expression) is coincident with a marked reduction in cytokine secretion, but no overall change in degranulation, in response to cross-linking of NK cell receptors or CD16 receptors. By contrast, increasing CD57 expression correlates with a gradual loss of responsiveness (in terms of CD25 expression, IFN-*γ* release and degranulation) to exogenous IL-12 + IL-18.

### Vaccine-driven, cytokine-mediated NK cell IFN-*γ* responses are dominated by the CD56^dim^ CD57^−^ and CD56^dim^ CD57^int^ NK cell subsets

**Figure 5:**
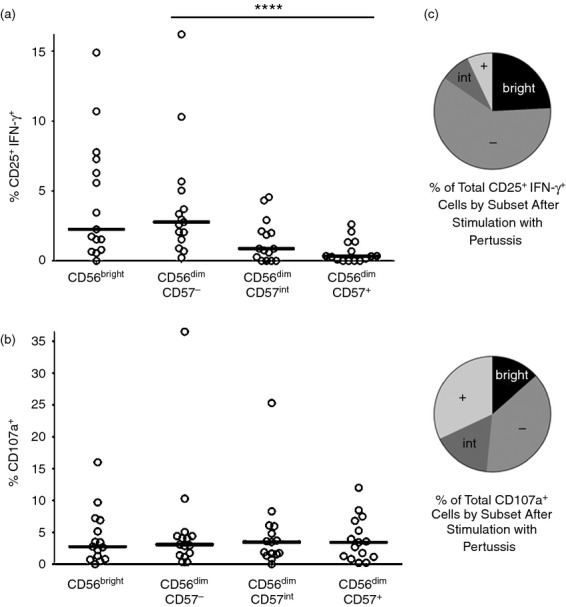
Natural killer (NK) cell interferon-*γ* (IFN-*γ*) responses to pertussis are dominated by the CD56^dim^ CD57^−^ and CD56^dim^ CD57^int^ NK cell subsets. Peripheral blood mononuclear cells were cultured with pertussis for 18 hr. The percentage of cells in each subset that are CD25^+^ IFN-γ^+^ (a) and/or CD107a^+^ (b) is shown. Each data point represents one donor, *n *= 14. CD56^dim^ subsets were analysed for linear trend with a repeated measures analysis of variance. ***P < 0·001. (c) Mean subset distribution of all IFN-*γ*^+^ NK cells (upper pie chart) and all CD107a^+^ NK cells (lower pie chart) for all donors (*n *= 14) shown in (a) and (b).

Accessory cytokines (including IL-12 and IL-18) and T-cell-derived IL-2 are known to be essential to drive NK cell IFN-*γ* responses during re-stimulation with vaccine antigens.^3^ Given that increasing CD57 expression correlates with loss of responsiveness to HCC, we predicted that CD56^dim^ CD57^−^ or CD56^dim^ CD57^int^ NK cell populations would show stronger ‘recall’ responses to whole cell pertussis than would CD56^dim^ CD57^+^ NK cells. To test this hypothesis, responses to pertussis (Fig. 1) were analysed for each of the four NK cell subsets defined by CD56 and CD57 expression (Fig. 5). There was a clear hierarchy of responses with a significantly higher proportion of CD56^dim^ CD57^−^ NK cells than CD56^dim^ CD57^int^ or CD56^dim^ CD57^+^ NK cells co-expressing CD25 and IFN-*γ* (*P *< 0·001 for linear trends) (Fig. 5a). On the other hand, CD107a expression was similar among all three CD57-defined NK cell subsets (Fig. 5b). When considering the proportion of all NK cells belonging to each subset together with the responsiveness of each individual subset, it became evident that vaccine antigen-driven NK cell IFN-*γ* recall responses occur almost entirely within the CD56^bright^ and CD56^dim^ CD57^−^ NK cell subsets with minimal contributions from the CD56^dim^ CD57^int^ and CD56^dim^ CD57^+^ subsets (Fig. 5c).

### CD57 acquisition is associated with reduced expression of cytokine receptors IL-12R*β*2 and IL-18R*α*

**Figure 6:**
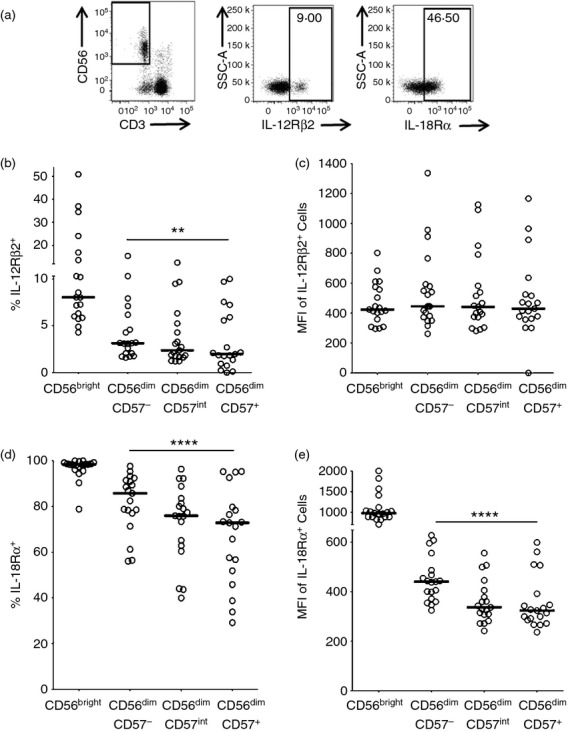
Interleukin-12 receptor *β*2 (IL-12R*β*2) and IL-18R*α* expression decrease with CD57 expression. Peripheral blood mononuclear cells were analysed *ex vivo* for IL-12R*β*2 and IL-18R*α* expression. (a) Representative flow cytometry plots for IL-12R*β*2 and IL-18R*α*. Frequency (b) and mean fluorescence intensity (MFI) (c) of IL-12R*β*2 expression, and frequency (d) and MFI (e) of IL-18R*α* expression, were assessed by subset. Each data point represents one donor, *n *= 19. Lines indicate median values. CD56^dim^ subsets were analysed for linear trend with a repeated measures analysis of variance. ****P ≤ 0·0001.

CD57 acquisition on NK cells is associated with a reduced ability to respond to accessory cytokines (Fig. 4) leading to a progressive decline in their ability to respond to vaccine-driven cellular responses by production of IFN-*γ* (Fig. 5a). To determine whether this is due to altered cytokine receptor expression and altered downstream signalling we assessed the resting (*ex vivo*) expression of IL-18R*α* and IL-12R*β*2 (Fig. 6). The proportion of IL-12R*β*2-expressing cells was highest among the CD56^bright^ NK cells with a progressive decrease in expression across the CD57-defined NK cell subsets (Fig. 6b) but IL-12R*β*2 expression density did not vary across subsets (Fig. 6c). Although IL-18R*α* was expressed at a much higher frequency than IL-12R*β*2 within all NK cell subsets, the same trend was seen, with declining IL-18R*α* expression, with increasing CD57 expression (Fig. 6d). In contrast to IL-12R*β*2, however, IL-18R*α* mean fluorescence intensity also declined with increasing CD57 expression (Fig. 6e).

## Discussion

Vaccination typically provides long-lasting protection against infectious diseases by inducing the expansion and differentiation of small populations of naive, antigen-specific, T and B cells into much larger populations of long-lived memory cells with enhanced effector function. In particular, antigen-specific memory CD4^+^ T cells augment B-cell, CD8^+^ T-cell and macrophage-mediated effector functions.^22^ Although circulating antibody may persist for many years after vaccination, frequencies of antigen-specific memory T cells are typically extremely low in peripheral blood (approximately 1 in 10 000^23^) and can be difficult to detect in the absence of recent boosting. However, the observation that IL-2 produced in an antigen-specific manner by CD4^+^ T cells can activate a substantial proportion (varying from ˜ 1% up to 60% in some cases) of all circulating NK cells,^2^,^3^,^13^,^18^,^24^ and that these responses can be detected for more than 20 years after vaccination in the case of DTP, suggests that NK cell responsiveness might represent a more sensitive biomarker of T-cell induction and maintenance and might therefore have a role to play in evaluation of new vaccines or new vaccine formulations. Whether NK cells – activated by T-cell IL-2 or by cross-linking of Fc receptors (CD16) by immune complexes – play an important role as effectors of vaccine-induced immunity is as yet unknown but the speed with which they are activated (within 6 hr of exposure to the pathogen^3^) and the large number of potentially responding cells suggest that their role should be investigated.

Here, we observed that NK cell responses to pertussis were significantly greater in magnitude than responses to DT or TT, even though all three antigens would have been administered together during vaccination. A likely explanation for this is that the pertussis antigen is a whole cell preparation containing numerous ligands for pattern recognition receptors on macrophages and dendritic cells, leading to their secretion of IL-12 and IL-18, which is necessary to induce NK cells to secrete IFN-*γ* and become cytotoxic.^4^,^13^ Purified toxoids such as DT and TT lack such ligands and so, *in vitro* at least, NK cells can only be induced to respond in the presence of exogenous IL-12 and IL-18. *In vivo*. however, infection by live tetanus and diphtheria bacteria would presumably induce a strong accessory cell cytokine response. On the other hand, much stronger NK responses to pertussis than DT or TT were seen even in the presence of LCC, suggesting that whole cell pertussis may also induce a stronger T-cell response than does a toxoid antigen.

**Figure 7:**
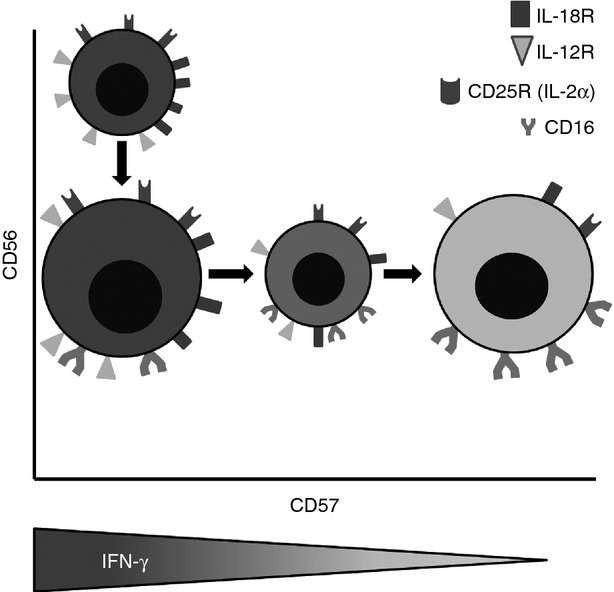
The relationship between natural killer (NK) cell phenotype and functional responses to exogenous cytokines. NK cell subsets defined by CD56 and CD57 expression also differ in their expression of interleukin-18 receptor *α* (IL-18R*α*), IL-12R*β*2 and CD16 and in their ability to up-regulate CD25. Declining expression of cytokine receptors with increasing expression of CD57 results in gradual loss of the ability of the cells to secrete interferon-*γ* (IFN-*γ*) after cytokine stimulation. The diameter of each cell reflects the approximate proportion of the entire NK population belonging to that subset. The shading of each cell reflects the capacity of that subset to produce IFN-*γ* in response to high-dose IL-12 + IL-18 (darkest shading denotes the highest IFN-*γ* production). NB: Our data indicate that CD107a expression does not differ significantly between subsets, whether induced by exogenous cytokines or receptor cross-linking.

Despite an overall tendency for NK cells to respond to vaccine antigens, there was considerable heterogeneity between individuals, which may in part be explained by inter-individual variation in T-cell IL-2 responses. However, we also observed heterogeneity between NK cell subsets in their responsiveness to vaccine-driven signals, with responses being dominated by CD56^bright^ CD57^−^ and CD56^dim^ CD57^−^ NK cells. This correlated with higher levels of CD25 expression on IL-12/IL-18-activated CD57^−^ cells compared with CD57^+^ cells and a higher resting level expression of IL-12R*β*2 and IL-18R*α* on these cells. The relationship between NK cell phenotype and responsiveness to exogenous cytokines is summarized in Fig. 7. These findings are in line with previous reports that CD57^+^ NK cells are less able to respond to cytokines,^10^,^12^ and express lower levels of IL-18Rα and lower amounts of mRNA for IL-12R*β*2, compared with CD57^−^ NK cells. IL-18 is known to induce expression of the high-affinity IL-2R*α* (CD25) on NK cells^25^ whereas IL-12 is necessary, but not sufficient, for their production of IFN-*γ*.^26^ Moreover, IL-2 induces expression of the inducible chain of the IL-12R (IL-12*β*2).^27^ Thus, as shown here, synergy between these three cytokine signals, IL-2, IL-12 and IL-18, results in NK cells producing high levels of IFN-*γ* during the first 18–24 hr following re-exposure to vaccine antigens.

Interestingly, we have observed that the maturation of NK cells from CD56^bright^ CD57^−^ to CD56^dim^ CD57^+^ is a gradual process with functional changes being highly correlated with CD56 and CD57 expression. This is particularly apparent for the cytokine-driven pathway of NK cell activation where expression of IL-12R and IL-18R as well as IL-12/IL-18-induced CD25 expression and IFN-*γ* synthesis are all very tightly negatively associated with CD57 expression. We find that CD57^int^ NK cells make significant amounts of IFN-*γ* after stimulation with high-dose IL-12/IL-18 but respond less robustly to low concentration cytokines and vaccine antigens, suggesting that they may fail to compete effectively with CD57^−^ NK cells when cytokines are limiting.

An area of increasing concern in industrialized countries is the burden of infectious disease and poor response to vaccination in the elderly population.^28^ Although ageing in the innate immune system, including age-associated changes in the composition, phenotype and function of circulating NK cells, is being linked to increased susceptibility to *de novo* viral and bacterial infections,^29^ deterioration of antigen-specific memory responses and reduced responsiveness to vaccination with increasing age tend to be attributed to narrowing of the T-cell repertoire and functional senescence of the T-cell pool.^30^,^31^ Our data suggest, however, that these two components of immune ageing may interact; deteriorating CD4^+^ T-cell responses will limit the availability of IL-2 to drive NK cell responses while, at the same time, the proportion of CD57^−^ NK cells able to respond to IL-2 will decrease. We predict, therefore, that vaccination-induced NK cell IFN-γ responses could decline with increasing age, potentially contributing to reduced vaccine efficacy in elderly populations. In addition, subclinical human cytomegalovirus (HCMV) infections may potentiate the functional differentiation and senescence of NK cells.^9^,^32^–^35^ Given that at least 40% of the world population is HCMV seropositive, and prevalence can exceed 95% in some African and Asian populations,^36^ HCMV exposure may contribute significantly to poor vaccine efficacy at a population level. Studies to test these various predictions are currently underway in our laboratory.

## Abbreviations

APC: allophycocyanin
DT: diphtheria toxoid
DTP: diphtheria–tetanus–pertussis
HCC: high concentration of cytokines
HCMV: human cytomegalovirus
IFN-*γ*: interferon-*γ*
IL-2: interleukin-2
LCC: low concentration of cytokines
NK: natural killer
PBMC: peripheral blood mononuclear cells
PE: phycoerythrin
rh: recombinant human
TT: tetanus toxoid
